# Clinical Presentation and Management of Swyer Syndrome: A Case Report

**DOI:** 10.7759/cureus.100735

**Published:** 2026-01-04

**Authors:** Yassine Errahali, Mohamed Malad, Ikhlass Lakssir, Jade Issouani, Ahmed Anas Guerboub

**Affiliations:** 1 Endocrinology and Diabetology Department, Mohammed V Military Academic Hospital, Rabat, MAR; 2 Faculty of Medicine and Pharmacy, Mohammed V University Souissi, Rabat, MAR

**Keywords:** case report, disorders of sex development, gonadal dysgenesis, primary amenorrhea, swyer syndrome

## Abstract

A pure 46, XY gonadal dysgenesis, or Swyer syndrome, is an extremely rare condition causing primary amenorrhea. It is distinguished by the existence of a female phenotype with a 46, XY karyotype. Reports of new cases are essential to improve early diagnosis and management.

We present a case of a 16-year-old Moroccan girl with pubertal delay. The patient exhibited a female, partially developed morphology with Tanner stage 2 secondary sexual characteristics, and a hormonal profile consistent with hypergonadotropic hypogonadism. Morphological evaluation revealed the presence of a uterus and vaginal cavity without visualization of the ovaries. Chromosomal analysis confirmed the diagnosis of Swyer syndrome. A laparoscopic operation revealed bilateral striated gonads, which had to be removed because of the risk of malignancy. Following the operation, estrogen-only pubertal induction was initiated, followed later by progesterone.

The case highlights the importance of considering Swyer syndrome in adolescent girls presenting with primary amenorrhea and delayed puberty. Our report features atypical characteristics, including partially spontaneous pubertal development and pronounced short stature, which may delay both diagnosis and treatment. Nevertheless, earlier diagnosis and comprehensive management, including early gonadectomy, hormone replacement therapy, and psychological support, remain essential for optimal outcomes.

## Introduction

Disorders of sex development (DSD) were historically described based on external phenotype, with terms that are now outdated and stigmatizing (male pseudohermaphrodite, female pseudohermaphrodite, true hermaphrodite, XX male, or XY sex reversal) [1]. The Chicago Consensus (2006) proposed a neutral classification based on karyotype, dividing DSD into three groups: 46, XX; 46, XY; and sex chromosome DSD [2]. Recently, a more precise pathophysiological understanding has been achieved through an etiological approach based on biological causes (abnormalities in gonadal determination, steroidogenesis, hormonal action, or internal organ development) [3].

Swyer syndrome is one of the rare forms of DSD. It is characterized by the presence of a 46, XY karyotype in a patient with external and internal female genitalia associated with hypergonadotropic hypogonadism [4]. Patients with Swyer syndrome may present delayed puberty and an increased risk of gonadal malignancies, particularly gonadoblastoma [5]. This case report describes specific features not often highlighted: partial pubertal development, severe stature delay, and diagnostic challenges in a limited-resource context.

## Case presentation

**Figure 1:**
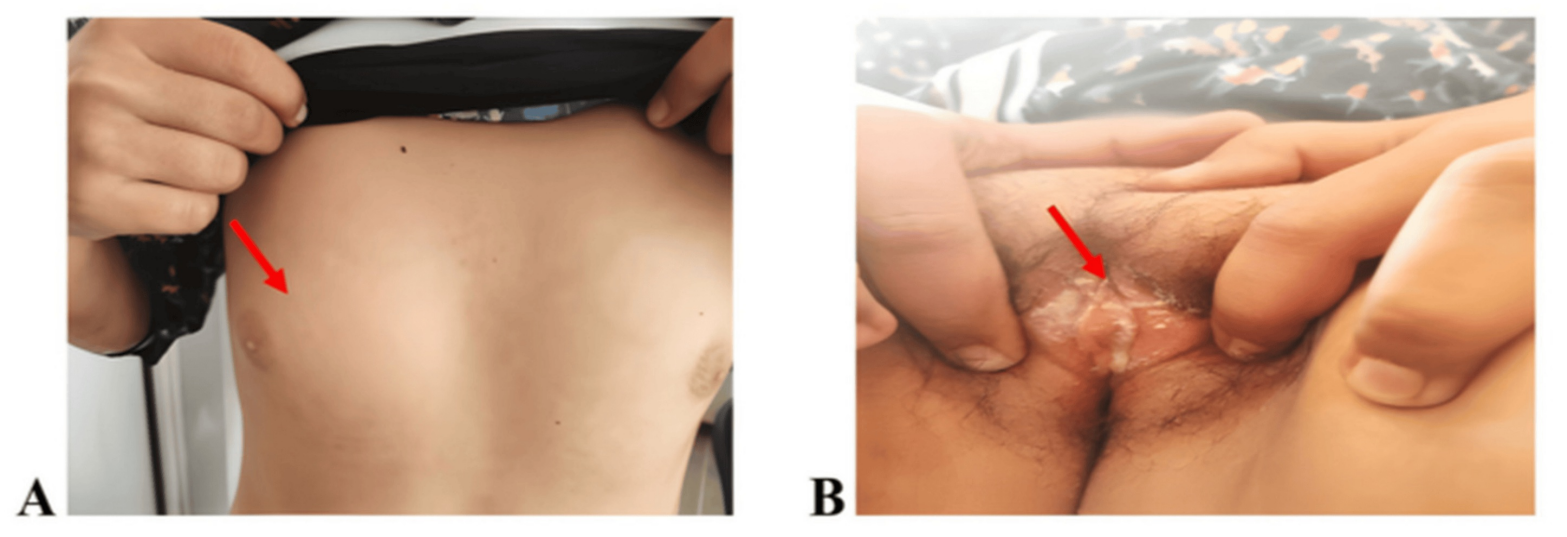
Pictures of the patient's breast development (A) and external genitalia (B) The red arrow shows the patient's breast development (Tanner stage 2) in panel A and typical female external genitalia with sparse pubic hair (Tanner stage 2) in panel B.

We report the case of a 16-year-old girl who was referred to our department for assessment of delayed puberty and primary amenorrhea. No significant medical or family history was identified. Her weight was 44 kg, corresponding to three standard deviations (SDs) below the mean for age, and her height was 147 cm, corresponding to −3 SD for age and −3 SD for target height. The patient had a female phenotype, with underdeveloped breasts (Tanner stage 2) and sparse pubic hair (Tanner stage 2) (Figure 1).

Examination of the external genitalia revealed normal female genitalia, with an intact hymen. Pelvic ultrasound and MRI confirmed the presence of a normal vaginal cavity, with the uterus measuring 60x24 mm and a normal myometrial signal; however, both ovaries were not visualized.

**Figure 2:**
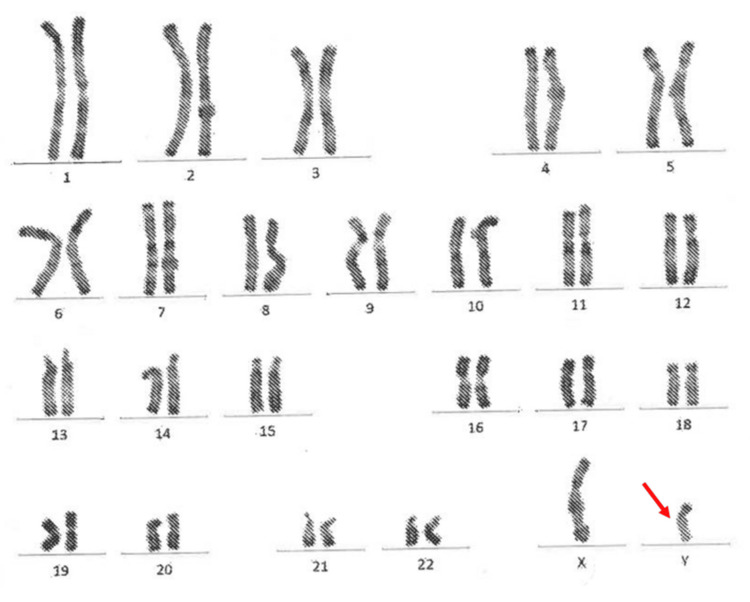
Patient's karyotype showing normal male 46, XY karyotype The red arrow indicates the presence of a normal Y chromosome in the patient’s karyotype.

Biological tests showed elevated levels of follicle-stimulating hormone (69.9 mUI/mL) and luteinizing hormone (12.6 mUI/mL), with low estradiol levels (20.3 pg/mL). Testosterone levels were also low (0.28 ng/mL). Anti-Müllerian hormone (<0.01 ng/mL) and inhibin B (<15 pg/mL), markers of Sertoli and granulosa cell activity, were significantly reduced, confirming the diagnosis of gonadal dysgenesis by proving the absence of functional gonadal tissue. A karyotype analysis was performed twice, confirming a typical male karyotype of 46, XY (Figure 2).

Regarding statural and weight delay, bone age was delayed (11 years and 6 months according to the Greulich and Pyle method) [6]. Investigations excluded chronic disease, malabsorption, endocrine disorders, or nutritional deficiencies. Growth hormone (GH) deficiency was also excluded, with normal insulin-like growth factor-1 levels for age (225 ng/mL) and a normal GH stimulation test (GH peak of 38 mIU/L in a combined glucagon/propranolol test). The delay in this context was attributed to a constitutional cause, aggravated by a sex hormone deficiency. The patient’s laboratory results are summarized in Table 1.

**Table 1:** Patient’s laboratory results with reference ranges AMH: Anti-Müllerian hormone, FSH: Follicle-stimulating hormone, GH: Growth hormone, IGF-1: Insulin-like growth factor-1, LH: Luteinizing hormone.

Parameter	Patient value	Reference range
FSH	69.9 mUI/mL	1.5–12.4 mUI/mL
LH	12.6 mUI/mL	1.7–8.6 mUI/mL
Estradiol	20.3 pg/mL	30–400 pg/mL (follicular)
Testosterone	0.28 ng/mL	0.1–0.8 ng/mL (female)
AMH	<0.01 ng/mL	1–4 ng/mL
Inhibin B	<15 pg/mL	15–300 pg/mL
IGF-1	225 ng/mL	190–429 ng/mL
GH stimulation	38 mUI/L (peak)	>20 mUI/L (normal)

A laparoscopic investigation revealed the presence of a small uterus, bilateral streak gonads, and fallopian tubes that appeared normal. Given the risk of malignant degeneration, bilateral laparoscopic gonadectomy and salpingectomy were performed. The pathological analysis of the samples found a dysgenic gonad without any signs of malignancy. Estrogen-only therapy was started initially for pubertal induction; progesterone was introduced later according to guidelines. Psychological support, a key part of the management of DSD, was provided as part of multidisciplinary care. Long-term follow-up was planned, but unfortunately, the patient was lost to follow-up.

## Discussion

Swyer initially documented the presence of pure gonadal dysgenesis 46, XY in two female subjects in 1955 [7]. The two women presented with primary amenorrhea, female external genitalia, and normal Müllerian tissues. It is estimated that approximately 1 in 80,000 live births are affected by this rare condition [8]. A mutation in the sex-determining region Y (*SRY*) gene, which is located on the distal short arm of the Y chromosome, can result in pure gonadal dysgenesis 46, XY (Yp11.3) [9]. In addition, pure XY gonadal dysgenesis can also result from other mutant genes, in particular *SOX9*, *DAX1*, *SF1*, and *WT1*. Such genes are implicated in the control of *SRY* expression or function as downstream transcriptional activators of *SRY* in the testicular determination pathway [10]. Unsuccessful testicular development leads to the formation of undifferentiated striated gonads, which are unable to produce androgens or anti-Müllerian hormone [9]. These genes were not tested in our case due to their very limited availability.

People with primary amenorrhea and absent or incomplete secondary sex characteristics are generally detected in their adolescence or early adulthood. The clinical presentations of Swyer syndrome are wide-ranging, and in the early stages, affected patients may have no obvious symptoms, which may delay diagnosis [11].

The treatment of pure 46, XY DSD typically involves preventive gonadectomy, adequate hormone replacement therapy, and psychological support. Gonadal crest tumors, including dysgerminomas, choriocarcinomas, gonadoblastomas (which occur in 30% of cases), and yolk sac tumors, are more prevalent in these patients. For these patients, an early gonadectomy is recommended as a means of reducing the likelihood of malignant gonadal changes. Psychological support is crucial, and disclosure should be appropriate for the patient's age and level of comprehension. Individuals with Swyer syndrome who are raised as females generally maintain a stable female gender identity. Fertility options, if desired, may include donor oocytes combined with assisted reproductive technologies [12]. In the present case, the patient underwent laparoscopic bilateral gonadectomy at the time of diagnosis before the initiation of hormone replacement therapy. Psychological support was provided by the psychiatric team at the hospital, with progressive disclosure of the diagnosis, including genetic sex, in compliance with adolescent counseling guidelines. Discussions also covered infertility and possible alternatives, such as adoption, given that oocyte donation is not possible in Morocco.

## Conclusions

The presence of primary amenorrhea associated with hypergonadotropic hypogonadism and the absence of visible ovaries on medical imaging justify considering Swyer syndrome as a potential diagnosis. Only a chromosomal analysis can establish a definitive diagnosis. This case also highlights several atypical characteristics, including partial pubertal development and significant growth retardation. Management should include long-term hormone replacement therapy, preventive bilateral gonadectomy, and psychological support. This requires coordinated care from a multidisciplinary team, including pediatricians, endocrinologists, surgeons, and psychiatrists.
